# To vaccinate or not to vaccinate? Experiences of COVID-19 vaccine uptake among people living with non-communicable diseases in Ghana: A qualitative study

**DOI:** 10.1371/journal.pgph.0003820

**Published:** 2024-10-14

**Authors:** Leonard Baatiema, Sheba M. P. Kunfah, Olutobi A. Sanuade, Luke N. Allen, Seye Abimbola, Ama de-Graft Aikins, Kwadwo A. Koram, Margaret E. Kruk

**Affiliations:** 1 Department of Health Policy, Planning and Management, School of Public Health, University of Ghana, Legon, Accra; 2 Department of Global Health and Population, Harvard T.H. Chan School of Public Health, Harvard University, Boston, Massachusetts, United States of America; 3 Department of Population Health Sciences, Spencer Fox Eccles School of Medicine, University of Utah, Salt Lake City, Utah, United States of America; 4 Department of Clinical Research, Faculty of Infectious and Tropical Diseases, London School of Hygiene and Tropical Medicine, London, United Kingdom; 5 School of Public Health, Faculty of Medicine and Health, University of Sydney, Australia; 6 Institute of Advanced Studies, University College London, London, United Kingdom; 7 Noguchi Memorial Institute for Medical Research, University of Ghana, Legon, Ghana; PLOS: Public Library of Science, UNITED STATES OF AMERICA

## Abstract

Following the development of a vaccine for COVID-19, the expectation was instantaneous widespread distribution and uptake to halt further spread, severe illness and deaths from the virus. However, studies show very low uptake, especially in resource-poor settings, and little is documented about the drivers of vaccine uptake in populations classified as high-risk. In this study, we explored access and uptake of COVID-19 vaccines among people living with non-communicable diseases (PLWNCDs) in Ghana. A qualitative study using in-depth interviews and focus group discussions was conducted among adults (>18 years) PLWNCDs stratified by sex, age, and type of non-communicable diseases (NCDs) at the community level (non-users of the health service) and health facility levels. Purposive sampling was used to select eligible participants. Topic guides were used to facilitate the face-to-face in-depth interviews and focus group discussions. The interviews and discussions were all digitally audio recorded. All transcripts and field notes were thematically analysed. Overall, 62 participants were recruited for this study. Family members, friends/peers, health workers and media were identified as the main sources of information for COVID-19 vaccines. Several barriers that mediated access to the COVID-19 vaccines in Ghana were reported including mistrust of vaccine efficacy and fears of vaccine side-effects, long distance to and waiting hours at vaccination centres, shortages of vaccines at vaccination centres and non-prioritization of NCD patients for the vaccine. To improve uptake, intensified education and sensitization, house-to-house vaccination, expansion of vaccination centers and increased supply of vaccines were recommended by participants. Compared to studies elsewhere, misinformation and disinformation were not major causes of vaccine hesitancy. If policymakers can improve community-based vaccine delivery, reduce queues and waiting times, prioritize PLWNCDs and other vulnerable groups, and improve sensitization and communication–our findings suggest there will be major improvements in COVID-19 vaccine coverage in Ghana.

## Background

Globally, cases of COVID-19 are declining, population immunity is increasing, restrictions are easing up and many countries are recovering from the unprecedented and enormous impact that COVID-19 had on health and all other aspects of life [1]. In early May 2023, the World Health Organisation declared that COVID-19 was no longer a public health emergency of international concern by [1]. However, COVID-19 remains a global health threat as new strains of the virus keep emerging [1]. People with non-communicable diseases (NCDs) suffered some of the worst outcomes in terms of morbidity and mortality [2–5]. The interplay between COVID-19 and NCDs presents different and severe effects to PLWNCDs according to several studies. For example, it was earlier reported that people with pre-existing NCDs (e.g. diabetes, hypertension, heart diseases, etc.) presented more severe and complications of COVID-19. This population also reported an increased risk and severity of COVID-19 [3], thus placing PLWNCDs as an important population of public health and policy interest. In Italy for instance, about 97.1% of patients who died from COVID-19 had one or more comorbidities with the majority being NCDs such as hypertension, type 2 diabetes, (Coronary Pulmonary Diseases (COPD), and cancers among others [6]. In Ghana, 38.9% of the COVID-19 cases reported in two of the major treatment centres had comorbidities in the form of NCDs; and the higher the number of comorbid conditions, the greater the severity of COVID-19 infection [4]. The WHO Rapid Surveys which reported on the extent of service disruptions during the pandemic also highlighted the extent to which PLWNCDs were massively affected and needed more protection [7].

The discovery of vaccines brought hope and contributed greatly to a major break in transmission and the occurrence of severe disease and death [8]. It was recently reported that the globally approved COVID-19 vaccines have a high level of vaccine effectiveness among real-life populations [9]. Initially, the main issue was securing adequate doses of vaccine, however as supply increased over time, vaccine hesitancy has become a more important global health challenge [10, 11]. The unwillingness of people to receive the COVID-19 vaccine is a huge threat to the attainment of targeted vaccine coverage [11]. There are varying acceptance rates across different countries and population groups [12]. Some studies across the world have shown that most of the people who hesitated were concerned about the side effects of the vaccine and its safety [10, 13], while others were also concerned about the short duration of the development of the vaccine, costs, and vaccine effectiveness. Countries such as China, the UK, Brazil, South Africa and the USA had acceptance rates above 80% while countries like the United Arab Emirates had acceptance rates as low as 22% [11]. In a study carried out in Nigeria, People Living With NCDs (PLWNCDs) were found to be hesitant about taking the vaccine with reasons that there may be adverse effects such as worsening of their conditions, concerns about the short duration for the manufacture of the drug, misinformation about the vaccine being meant for people who already have the disease and the issues of mistrust of the manufacturers, government, and vaccine efficacy [14].

Ghana made history as the first country to receive 600,000 doses of the COVAX AstraZeneca/Oxford vaccine on the 24th of February 2021, but has not been left out in the battle against vaccine hesitancy in the general population [15]. A study that assessed vaccine acceptability across three regions in Ghana found that vaccine acceptability was generally low. Vaccine acceptability rates were 32.5%, 29.6% and 26.2% in the Ashanti, Western North and Northern regions respectively. There was an overall vaccine acceptability of 41.9% and factors such as the educational level of the individual, being employed and recently receiving a previous vaccine were positively associated with vaccine acceptability [16].

To ensure successful vaccination of the population, the government developed measures and priority guidelines to guide the vaccination process. The following population groups were prioritized namely: health workers, people aged 60 years and above, persons with underlying conditions, among others [17]. To complement these, efforts were made to create awareness and acceptance of the vaccine through health promotion and education activities in traditional and social media. This was important to counter existing myths, misperceptions, and lack of accurate information about the vaccines [17, 18]. Such activities were also tailored toward particular populations (community messaging of COVID-19 19 vaccine uptake benefits, etc). Ghana set a target of vaccinating about 20 million people or at least 60% of the population with the strategy to cover at least 20% of the target population by September 2021 and reach 60% by the end of 2021 per the targets set for all African Union (AU) members [15]. However, as of 4th June 2023, about 22,384,226 vaccine doses (WHO, 2023) have been administered and only 32.7% of the population have completed their primary series and 13.1% have had an additional dose or a booster [19]. This suggests that amid other vaccine challenges, vaccine hesitancy is a major barrier to attaining the targeted coverage. It is important to address vaccine hesitancy in the general population as well as population groups with high risk of severity and complications to improve vaccine coverage [3, 5, 11, 13, 14, 20]. In this study, we aimed to explore the perspectives of PLWNCD on the COVID-19 vaccine and their concerns about the vaccine. We envision that our findings will help equip policymakers as they formulate targeted strategies as efforts are being intensified at global and local levels to widen the coverage of COVID-19 vaccines, especially among people at high risk of severe illness and death from the COVID-19 virus.

## Methods

We adhered to the Standards for Reporting Qualitative Research (SRQR) guideline, [21] to elucidate the findings on the experiences of COVID-19 vaccine uptake among people living with NCDs in Ghana.

### Study sites

The study was conducted in three regions of Ghana: one region in each of the three zones (Northern, Middle and Coastal) to ensure representation of the different ecological zones of the country. For each zone, we purposively selected one region with a major healthcare referral center. For this reason, the regions considered for this study comprised the northern region, Ashanti region and Greater Accra region. Each of these regions had major referral/tertiary-level facilities for the screening, treatment, and management of COVID-19 and NCDs. The study sites from these zones were geographically, and socially and culturally diverse, with different socio-economic development trajectories in Ghana.

### Design

A qualitative study design was employed using two qualitative methods: in-depth interviews and focus group discussions with PLWNCDs. This design is methodologically flexible and allows participants to discuss, engage and explore the topic to generate new and contextually relevant, and rich insights on the topic. This study was embedded within a larger study that sought to redesign health systems in Ghana to provide better care for PLWNCDs during future public health shocks. The reporting of this study followed the Standards for Reporting Qualitative Research.

### Study participants, sampling and recruitment

To ensure maximum variation in the inclusion of participants for the study, in-depth interviews were conducted among adults (>18 years) PLWNCDs stratified by sex, age, and type of NCDs.

A purposive sampling method was used to select eligible participants for the in-depth interviews and focus group discussions. The sampling technique aimed to recruit participants in relation to the years of living with NCDs to ensure the study captures a wide and diverse breadth of experiences and perspectives on vaccine uptake. The sample size was determined based on sample saturation, that is, recruitment and interviews continued until a wide diversity of the issues were captured.

Recruitment of participants was done at two levels using different techniques and approaches. First, we conducted exit interviews: participants were recruited through engagement with the health facility authorities, unit heads, and health workers in consulting rooms. They were briefed about the study and invited to participate in the study after consultation. Secondly, at the community level, we identified and recruited PLWNCDs through the assembly members (community representatives) in collaboration with the Ghana NCDs Alliances who have established and supported networks of people living with NCDs. We leveraged the existing relationship between participants and the Ghana NCDs Alliance and their trust in the Assemblymen to recruit them into the studies. Overall, PLWNCDs who had met the above criteria, and were willing to participate, with written informed consent obtained, were eligible for the study.

### Interview guide

An interview guide was used to facilitate the discussions and conversations for the face-to-face in-depth interviews and the focus group discussions. The content of these guides was informed by an extensive review of the literature on COVID-19 vaccine uptake and experiences. The guide asked questions about their vaccination status, and reasons for vaccine uptake or non-uptake, exploring participants’ views on any potential hesitancy, trust, perceptions of risk, efficacy, safety, or perceived benefits or risk of the COVID-19 vaccine. The questions also explored their access to the vaccines, barriers to access, and recommendations to improve access and uptake. Details of the interview guides are provided in S2 Appendix.

### Data collection

To ensure there was consistency in the data collection procedures, topic guides were utilized to facilitate the face-to-face interviews and focus group discussions. Data collection was conducted by the lead researcher and trained six research assistants with prior experience in conducting qualitative in-depth interviews and focus group discussions among PLWNCDs. The interviews were conducted in the English Language and three other Ghanaian local languages namely Ga, Twi, and Dagbani before translation and transcription into the English Language. Three pilot interviews were conducted to test the clarity, appropriateness, relevance, and completeness of the topic guides and patients’ understanding of the questions prior to the data collection. The pilot exercise confirmed the appropriateness and comprehensiveness of the topic guide and as a result, the results were added in the final analysis. We conducted 38 face-to-face interviews at various health facilities after patient consultation visits. The interviewers used probes to explore for further insights from participants on the question or topic.

Four FGDs were conducted at the community level (comprising 5–6 members per session) targeting those not likely to access health services. All FGDs and interviews were audio-recorded using a digital voice recorder. The interview and group discussions proceeded until the researchers reached saturation. To confirm saturation, two additional interviews were conducted but these interviews were not added to the final analysis since they added nothing new to what was previously collated. Detailed field notes were also taken before, during and after the interview process to capture all relevant data that the audio recordings cannot. The content from the field notes aided the analysis and interpretation of the findings Overall, each interview lasted for about 40 minutes.

### Data analysis

All interviews were recorded and transcribed verbatim. For interviews conducted in the local language, they were transcribed from the local language to English language. All interview transcripts and field notes were thematically analyzed. The analysis followed several steps. First, all transcripts from the audio recordings of interviews and group discussions were edited and quality-checked to improve comprehension. Following a review of eight transcripts, a codebook was developed by the lead researcher which outlined the main themes and sub-themes. The codebook was developed based on the emergent categories from the transcripts and the study aims and interview guides. These were subsequently reviewed and adapted following the emergence of new themes and subthemes. Sample quotes from participants and operational definitions of the main themes were also presented in the codebook. All transcripts were imported into MAXQDA, a qualitative software package used to manage the qualitative data coding and analysis process. All transcripts were critically reviewed and coded independently by a second coder. A thematic content analysis was thus conducted according to the study aims and reported narratively and textually. Selected quotes from participants were used to highlight key themes.

### Trustworthiness

To enhance trustworthiness, the entire data collection process was supervised by the lead author who also participated in the data collection process in the field. The authors are experienced in conducting qualitative research and thus provided sufficient oversight before, during, and after the data collection/analysis. All interviews were audio-recorded and transcribed verbatim. Pilot interviews were conducted, and this helped ensure participants understood the questions and provided appropriate responses to answer the study questions. To check the quality of the data that were transcribed, the lead researcher resampled and reviewed a few of the transcripts by matching them with the audio recordings and the interview guide. However, transcripts were not returned to the interviewees and no interviews were repeated for cross-checking and validation.

### Inclusivity in global research

Additional information regarding the ethical, cultural, and scientific considerations specific to inclusivity in global research is included in the Supporting Information (S1 Appendix).

## Results

This section reports the findings from both the qualitative in-depth interviews at the health facility level and focus group discussions at the community level. Participants comprised of people living with NCDs such as stroke, hypertension, diabetes, and asthma. Overall, we noticed that both the vaccinated and the non-vaccinated had almost equal proportions of unvaccinated populations. Furthermore, for full vaccination (n = 10), the sample recruited from the health facility was slightly higher with 6. Most of the full vaccinations were for populations with hypertension or living with both hypertension and diabetes. Table 1 presents the characteristics of the participants and what follows next are the themes and emergent sub-themes.

**Table 1:** Characteristics of study participants.

Participant # ID	Sex	NCD Type	Study site	NCD Duration	Vaccination status	Vaccination dosage
1	Male	Diabetes	Health facility	5	Not vaccinated	Not applicable
2	Male	Hypertension	Health facility	12	Vaccinated	Full vaccination
3	Female	Diabetes and hypertension	Health facility	11	Not vaccinated	Not applicable
4	Male	Diabetes and hypertension	Community	15	Vaccinated	Full vaccination
5	Female	Hypertension	Health facility	17	Vaccinated	Partial (One shot)
6	Male	Diabetes	Community	22	Not vaccinated	Not applicable
7	Female	Diabetes and hypertension	Community	3	Vaccinated	Partial (One shot)
8	Female	Hypertension	Health facility	23	Not vaccinated	Not applicable
9	Female	Diabetes	Community	24	Vaccinated	Partial (One shot)
10	Male	Stroke	Community	24	Not vaccinated	Not applicable
11	Male	Hypertension	Health facility	23	Vaccinated	Partial (One shot)
12	Female	Hypertension	Community	22	Not vaccinated	Not applicable
13	Female	Diabetes	Health facility	21	Vaccinated	Full vaccination
14	Female	Hypertension	Health facility	1	Not vaccinated	Not applicable
15	Male	Diabetes	Health facility	4	Vaccinated	Partial (One shot)
16	Male	Diabetes, stroke and hypertension	Community	12	Not vaccinated	Not applicable
17	Male	Hypertension	Health facility	23	Vaccinated	Full vaccination
18	Female	Diabetes and hypertension	Health facility	20	Not vaccinated	Not applicable
19	Female	Diabetes	Health facility	15	Vaccinated	Partial (One shot)
20	Male	Diabetes	Health facility	17	Vaccinated	Partial (One shot)
21	Female	Hypertension	Community	9	Not vaccinated	Not applicable
22	Male	Diabetes	Health facility	11	Vaccinated	Partial (One shot)
23	Female	Stroke	Health facility	3	Vaccinated	Full vaccination
24	Male	Diabetes and hypertension	Community	7	Vaccinated	Full vaccination
25	Female	Diabetes	Health facility	15	Not vaccinated	Not applicable
26	Male	Diabetes, stroke and hypertension	Community	2	Vaccinated	Partial (One shot)
27	Female	Hypertension	Health facility	1	Not Vaccinated	Not applicable
28	Female	Diabetes	Health facility	12	Vaccinated	Partial (One shot)
29	Female	Diabetes and hypertension	Health facility	14	Vaccinated	Partial (One shot)
30	Male	Hypertension	Community	8	Not vaccinated	Not applicable
31	Male	Diabetes and hypertension	Health facility	9	Vaccinated	Partial (One shot)
32	Female	Hypertension	Health facility	16	Vaccinated	Partial (One shot)
33	Male	Diabetes and hypertension	Health facility	11	Vaccinated	Partial (One shot)
34	Female	Diabetes, stroke and hypertension	Community	3	Not vaccinated	Not applicable
35	Female	Hypertension	Health facility	8	Not vaccinated	Not applicable
36	Female	Diabetes	Health facility	9	Vaccinated	Partial (One shot)
37	Male	Stroke	Health facility	3	Not vaccinated	Not applicable
38	Female	Hypertension	Community	28	Vaccinated	Partial (One shot)
39	Female	Diabetes	Community	8	Not vaccinated	Not applicable
40	Male	Asthma	Health facility	10	Vaccinated	Partial (One shot)
41	Male	Hypertension	Community	5	Not vaccinated	Not applicable
42	Female	Diabetes	Health facility	1	Vaccinated	Partial (One shot)
43	Male	Diabetes	Community	17	Not vaccinated	Not applicable
44	Male	Hypertension and diabetes	Community	12	Not vaccinated	Not applicable
45	Female	Diabetes	Health facility	30	Vaccinated	Full vaccination
46	Female	Diabetes and hypertension	Community	15	Vaccinated	Full vaccination
47	Male	Hypertension	Community	16	Not vaccinated	Not applicable
48	Male	Diabetes and hypertension	Community	13	Vaccinated	Partial (One shot)
49	Female	Stroke	Health facility	1	Not vaccinated	Not applicable
50	Female	Hypertension	Health facility	15	Vaccinated	Full vaccination
51	Female	Diabetes	Health facility	14	Not vaccinated	Not applicable
52	Male	Asthma	Health facility	20	Vaccinated	Partial (One shot)
53	Female	Hypertension	Community	16	Not vaccinated	Not applicable
54	Female	Diabetes and hypertension	Health facility	24	Vaccinated	Partial (One shot)
55	Male	Hypertension	Health facility	19	Not vaccinated	Not applicable
56	Male	Diabetes and hypertension	Community	5	Vaccinated	Full vaccination
57	Female	Asthma	Health facility	15	Not vaccinated	Not applicable
58	Male	Hypertension	Health facility	10	Vaccinated	Partial (One shot)
59	Male	Diabetes and hypertension	Health facility	11	Not vaccinated	Not applicable
60	Female	Diabetes	Health facility	1	Vaccinated	Partial (One shot)
61	Male	Diabetes and hypertension	Community	2	Not vaccinated	Not applicable
62	Female	Hypertension	Health facility	4	Vaccinated	Partial (One shot)

### Summary of themes on access and uptake of the COVID-19 vaccines

**Fig 1:**
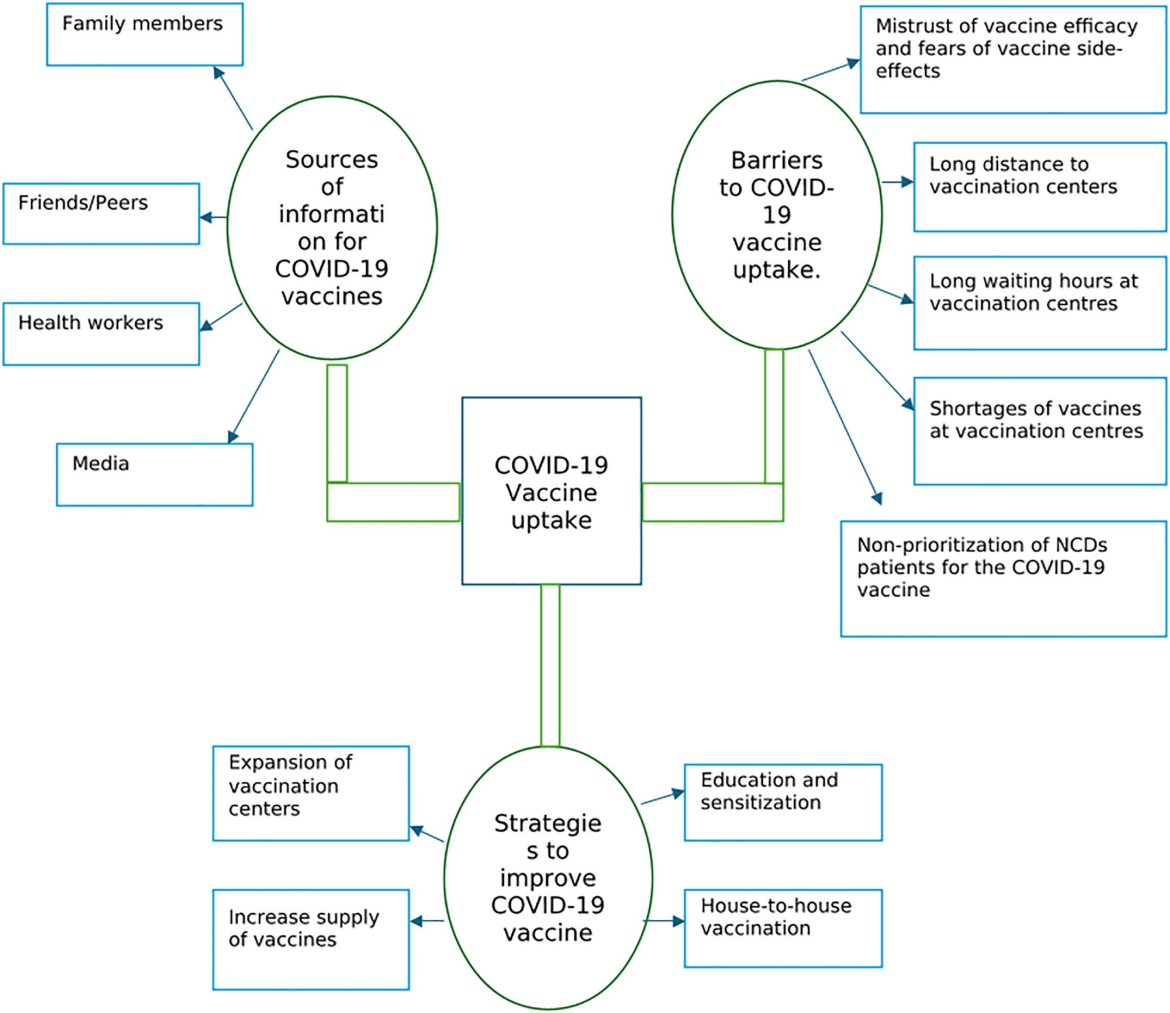
Access and uptake of COVID-19 vaccines among people living with NCDs.

Access to the COVID-19 vaccine among those who desired to take the shot was featured during the interviews and in the focus group discussions. First, the study showed that participants received information about the vaccine from four key sources namely family members, friends/peers, health workers and media. Several barriers mediated access to the COVID-19 vaccines in Ghana among PLWNCDs and these comprised mistrust of vaccine efficacy and fears of vaccine side-effects, long distance to vaccination centers, long waiting hours at vaccination centers, shortages of vaccines at vaccination centers and non-prioritization of NCDs patients for the COVID-19 vaccine. To improve access, participants suggested governments and stakeholders intensify their education and sensitization drive, through house-to-house campaigns, expansion of vaccination centers and increase in the supply of vaccines. Fig 1 provides an overview of the themes and sub-themes.

## Theme 1: Sources of information about COVID-19 vaccines

Here, we sought to understand from the participants, their sources of information about the COVID-19 vaccine. The analysis showed that the study showed that participants received information about the vaccine from four key sources namely family members, friends/peers, health workers and media.

### Sub-theme 1: Family members

Some of the participants reported that they received information about the COVID-19 vaccine from family members. According to some, although they got a lot of information about the vaccine from other sources, family members were the main source. Such family members were also healthcare workers whom they often call to confirm or get the latest information about the vaccine and the pandemic overall.

*Everyone had some information about the pandemic and the vaccine efficacy and issues of trust about the vaccine. For me, at the end of the day, I reached out to family members or relations who worked in the health sector. This is family so I trusted information coming from family members, especially those who either worked in the health sector or know someone working there.*

***(Participant # ID 20)*.**

### Sub-theme 2: Media

Almost all the participants noted the media as the main source of information about the vaccine. They acknowledged this was from multiple media sources but mainly through information on television and radio stations. Only a few reported receiving vaccine information from social media as they acknowledged a lot of misinformation and fake news about the vaccine efficacy were mainly from social media such as Facebook.

*Since the pandemic started, every information I receive and currently have about it has been from the media. I follow the news so I know what is happening about the vaccines, and where they get them although this can be misleading because we know the vaccines are not there and yet, they will report it in the news that we have vaccines available.*

***(Participant # ID 2)***

Participants also expressed concern about fake news and mistrust regarding vaccine availability and efficacy. They believed access to such information should be encouraged from reputable media houses. One participant had this to say,

*Though one needs to restrain some caution about the millions of information out there about the pandemic and the effects of these vaccines, we need to access information from very reputable media houses.*

***(Participant # ID 57)***

### Sub-theme 3: Health workers

Most of the participants also noted that health workers were the main source of information about the vaccine, especially information about the vaccine type to take, clarification about the side effects and where to receive the vaccine.

*Although I am yet to be vaccinated, I have a lot of information about the vaccine from my doctor. Before we enter the consulting room, the nurses come to educate us about the pandemic, the need to wear facemasks and the use of hand sanitizers. So, I got all my information about the vaccine and issues around vaccination. So, I know a lot through my doctors and nurses at the La Polyclinic.*

***(Participant # ID 25)***

Participants suggested that the health workers were a more reliable source of information about the COVID-19 vaccines. This view was expressed primarily from the participants who were recruited and interviewed at the health facility level.

### Sub-theme 4: Friends/peers

Information about the vaccine was also sought or received from friends and peers. According to some participants, they receive most of the information about the vaccine from friends or their peers at the workplace, during hangouts. This came up strongly from the focus group discussions where some shared how their friends were their main source of information about the vaccine, especially information about the side effects or perceived efficacy of the vaccines.

*Oh, most of us here I can say received information about the vaccine from our friends and peers. I mean the people we hang out with. Not sure about others but for me and same as some others here I believe, we meet almost every evening at our hangout venues and discuss these things. I get informed a lot about issues regarding the vaccine that I had no idea. My friend was convinced in one of such meetings to take the vaccine.*

***(Participant # ID 41)***

## Theme 2: Barriers to COVID-19 vaccine access and uptake

Participants in both the focus group discussions and the in-depth qualitative interviews discussed their experiences of barriers to accessing vaccines during the pandemic. Here, the questions in the topic guides explored their journeys in accessing and getting vaccinated, efforts to get vaccinated but unsuccessful, or reasons for their decision not to get vaccinated. The participants identified different challenges namely mistrust of vaccine efficacy and fears of vaccine side-effects, long distance to vaccination centers, long waiting hours at vaccination centres, shortages of vaccines at vaccination centers and non-prioritization of NCD patients for the COVID-19 vaccine.

### Sub-theme 1: Mistrust of vaccine efficacy and fears of vaccine side-effects

A dominant theme arising from this study was the participants’ experiences after taking the vaccine for COVID-19. These experiences mainly relate to feeling swollen arms, pain in the arms, fatigue, and headaches. These experiences of side effects were reported across the study participants and gender dispositions and NCD types. Participants were further probed to know if these side effects were of concern and made them regret their decision to take the vaccine. They unanimously noted that such side effects were normal and common with the introduction of the vaccination or medication into their body system. Some also mentioned that these side effects were expected, and it is something they read or heard about in the news and on social media, so they anticipated such experiences.

*A friend warned me that due to my diabetes condition, it was not safe for me to take the vaccine. This scared me and kept me away from going for the vaccine. I consulted other friends and family members who were also living with diabetes, and they repeated the same information to me.*

***(Participant # ID 43)***

We found that some of the participants were yet to be vaccinated and refused to get vaccinated due to the perceived side effects and an aggravated risk of complications from the shots for people with pre-existing chronic conditions. They were told that COVID-19 easily infected those with pre-existing chronic conditions, so they became weary of taking the vaccines. Another participant had this to say,

*As for the vaccine people are afraid because of what people have been saying about the vaccine. I also heard the same thing you get sick when you go for the injection. I think that has also scared a lot of us from going for it.*

*[* ***Participant # ID 18]***

### Sub-theme 2: Long distance to vaccination centers

Geographical access to vaccination centers was also observed to be a major deterrent to the COVID-19 vaccine uptake. This was observed among the study participants who opined that most of the vaccination centers were far and not within their reach. Although some vaccination centers were sited closed to the communities, they rarely had in stock the vaccines so most of them had to travel to other faraway centers to be able to access the COVID-19 vaccines. The challenge of long-distance to vaccination centers was unanimously shared among all the stroke survivors or their carers who expressed concern about how this challenge delayed the uptake of the COVID-19 vaccine.

*The challenge I had was the distance I had to travel to get vaccinated and the queue was unbearable. I got there in the morning, and I had to leave there around five in the evening just to get vaccinated.*

*[* ***Participant # ID 17]***

### Sub-theme 3: Long waiting time at vaccination centres

Participants expressed frustration about waiting for long hours to get vaccinated and this resulted in many abandoning the whole process or refusing to get vaccinated. Although they were interested in getting vaccinated, they could not fathom the experience of having to wake up in the early hours of the day to join queues, and even then, not be guaranteed a vaccination. These stories mainly emerged from people living with hypertension and diabetes who were keen on getting vaccinated due to their being classified as a priority group and at higher risk of COVID-19 complications. Whilst some centers provided chairs for people to sit and wait to get vaccinated, other centers did not have that, so people had to stand in the queue for long hours. Participants attributed these long hours to limited staff and inadequacy of vaccines, which were rolled out on a first-come, first-serve basis.

*Yes, I had to spend some hours waiting in a queue at Achimota Hospital. Though you will have to wait in queue for it to get to your turn, they did well by providing us with chairs to sit on while waiting to get vaccinated. You go to a center and you have just two people taking records and one person giving the injection so if you have about two hundred people coming to vaccinate you can only imagine when you will leave there*

***[Participant # ID 45]***

### Sub-theme 4: Shortages of vaccines

There was unanimity among participants in the in-depth individual interviews and the focus group discussions that stock-out and lack of vaccine supplies were major impediments to their motivation to access the vaccines. Participants expressed strong views regarding the news about the vaccine stock-outs that were repeatedly reported and discussed in the various media outlets (e.g. TV, radio, print media); this delayed and prevented them from trying to access the vaccines. Even in instances where announcements were made about the arrival of new supplies, they encountered long queues, or the vaccines were only available in centers remote from their communities and the centers located close to their communities lacked the vaccines. After several failed attempts to get the vaccines, participants moved on and felt reluctant to follow up and get the vaccines for COVID-19.

*They kept announcing that we should go for the shots and yet the vaccines were not accessible. See all the long queues and long distances people have to walk to travel to go get the shots. It is not worth it. I tried a few places and did not get my second shot even though the period to take the second shot had expired. Now should I start all over again?*

*(* ***Participant # ID 29)***

### Sub-theme 5: Non-prioritization of NCD patients for the COVID-19 vaccine

One of the major barriers to COVID-19 vaccine uptake, as identified by participants, was the lack of priority and importance given to PLWNCDs. Participants expressed disappointment about the government’s rhetoric of prioritizing PLWNCDs for the state vaccination program. Although participants earlier welcomed the state’s announcement to prioritize key populations such as the aged, health workers and people living with NCDs, their excitement was short-lived when the vaccination program was rolled-out and there were no institutionalized measures to ensure that key populations were targeted and prioritized for the vaccination roll-out.

*We are less important to them and that is why we are not given special attention for the vaccination strategy of government. Did you hear the president mention that we as people living with NCDs, aged, and health workers are key populations to be protected and prioritised when the vaccination starts? But what do we see? You go to the vaccination centers and everyone is treated equally and the officers don’t have any measure or information about people living with NCDs to prioritize us in the vaccination process. It is better to be home rather than go out to struggle for a vaccine when I have been told my risk level for COVID-19 is very high*

*(* ***Participant # ID 6)*.**

This, in their view, characterized the peak of the myriads of challenges they faced. For them, people with pre-existing chronic conditions needed to be prioritized and vaccinated first before the other groups to minimize their risk and exposure to infection and potential complications.

## Theme 3: Improving access to COVID-19 vaccines

Based on challenges relating to access to the COVID-19 vaccines, participants were asked about their views on measures to improve vaccine uptake and four thematic points were revealed, namely the need to intensify education and awareness creation in the communities, embark on house-to-house campaigns, and establish multiple vaccine centers and increase the supply of vaccines.

### Sub-theme 1: Education and sensitization

Intensification of education and community sensitization programs emerged as one of the key recommendations for two reasons. The first reason was to improve vaccine uptake and secondly, to demystify prevailing misinformation and misperceptions about the virus which proved to be counterproductive. In their views, such community awareness programs will play a catalytic role in educating community members about the need to get vaccinated and as a means to inform the public about the safety and efficacy of the vaccines since some people doubted the efficacy of the vaccines to prevent COVID-19 infection to minimize or prevent complications from the virus.

*I think in the radio and television adverts, they could have highlighted that people living with our condition are at high risk and so must take the vaccine. A lot of education needs to be done to encourage more people to take the vaccine. I think that the education should not stop. It should continue on radio, TV, and also at the hospitals.*

***[Participant # ID 32]***

Participants observed that misinformation and fear of vaccine side-effects were quite rife in the communities and thus recommended the rollout of massive community education and awareness creation about the safety of the vaccines to demystify current myths and information about the vaccines. For participants, these are the most critical issues underpinning their decision to vaccinate or not to vaccinate. That is, for any efforts to improve uptake, programs to promote community education and awareness are paramount.

*“in this community and maybe in others close by, we continue to express fears about the side effects of the vaccine, and this is deeply rooted in the La community and not just a perception so if the government is serious about the vaccine rollout, they need to create more awareness and carry out community engagement activities to address all the myths and fears associated with the vaccine.*

***[Participant # ID 44]***

### Sub-theme 2: House-to-house vaccination

To facilitate vaccinations, participants recommended the need for house-to-house campaigns to ensure that no one was left behind in the vaccination rollout. They observed that because of the perception that PLWNCDs were at a higher risk of COVID-19 complications, they believed several people were locked up in their rooms and houses due to the fear of the virus and needed to be reached through innovative campaigns and one of such campaigns was the house-to-house campaigns. This approach was common and used in past immunization programs with much success and did not fathom why a similar approach could not be instituted to improve the uptake of the COVID-19 pandemic.

*I think as they do with the polio vaccine moving from house to house, they can do the same with the coronavirus vaccine. Some people have heard about the vaccine but haven’t made up their minds to go for it. I think the house-to-house will enable the nurses to encourage people to take the vaccine.*

***[Participant # ID 31]***

*As I said earlier, they should go from house to house to get people vaccinated, people who cannot walk, sick people, and all that. They should help people, those who don’t have family to take care of, those who have stroke and diabetes, these patients who cannot afford to buy drugs for themselves and all that, I think the government should come to their aid and help them so that even if they are afraid of this covid, it will put some fear out of them and then they can get better.*

***[Participant # ID 44]***

### Sub-theme 3: Expansion of vaccination centers

Different views were expressed about the need to create multiple centers for vaccinations as they believed the existing centers were limited. A key suggestion was to create many vaccination centers at health centers, both private and public health facilities and at the community centers. One participant suggested the use of polling stations and voter registration centers as already established meeting points for national or local-level elections. These in his view were well-known centers by the community members and easy to rollout out the COVID-19 vaccination programme. This suggestion in their view would be instrumental in boosting vaccine uptake.

*If the vaccination is available at the hospitals where the PLWNCDS receive care, we will take it. But if we are told to go here; go there for the vaccine, it doesn’t encourage us to pursue this. the vaccination centers should be expanded. In fact, it can be located at health facilities in the community and private facilities as well so that people living with NCDs can easily go there for vaccination during their routine check-ups/reviews.*

***[Participant # ID 56]***

### Sub-theme 4: Increase the supply of vaccines

To encourage uptake of the COVID-19 vaccine, one key recommendation from participants from the focus group discussions and in-depth interviews was the supply of vaccines to ensure uptake and continuity to complete the full dosage. They expressed frustration about the shortages and stockouts at various vaccination centres and thus recommended the need for more vaccines to be distributed to the vaccination centres. They believed the shortages were one of the key factors for their decision not to go for the vaccine or complete their dosage.

*Look, even if you change your mind to go for the vaccine, you will still face accessibility issues. I see it in the news everyone about the lack of vaccines in the country. So, for me, the government should ensure there is an increase and regular supply of vaccines before they can encourage or call on us to get vaccinated.*

***[Participant # ID 18]***

## Discussion

There is a shred of clear evidence that vaccine hesitancy has been a major obstacle to global efforts to contain the COVID-19 pandemic [22]. Understanding and increasing COVID-19 vaccine uptake is critical to developing an effective post-pandemic strategy [23]. Our study explored barriers to COVID-19 vaccine uptake and recommendations for improving uptake among people living with NCDs in three regions of Ghana. Participants mentioned multi-level barriers to COVID-19 vaccine uptake including individual-level (fear of perceived side effects and risk of vaccine complications), healthcare system-level (long-waiting hours in vaccination centers) and structural-level (non-prioritization of people living with NCD and long distance to vaccination centers) barriers.

Some of the participants in our study were unvaccinated due to fear of perceived side effects and risk of complications of the COVID-19 vaccine, especially for people with pre-existing chronic conditions. This finding is consistent with findings from other settings in Ghana [24] and national surveys in the United Kingdom which showed that fear of vaccine side effects was a significant cause of vaccine hesitancy [23, 25]. Evidence from other contexts however showed that fear of COVID-19 contributed to vaccine hesitancy [26, 27]. The study by Rad et al. (2022) showed that even though fear of COVID-19 did not increase confidence in the vaccination in Iran, it was a motivating factor for individuals who otherwise might not get vaccinated at all [27]. Studies have further shown that fear generally stems from a lack of knowledge about COVID-19 or the COVID-19 vaccine indicating the need for healthcare providers to be transparent with patients in all aspects of care [28]. Hence, to minimize fear of vaccine uptake among our study population, healthcare providers need to distil and demystify evidence regarding the vaccine’s safety, efficacy, and clinical trial protocols, especially for people with pre-existing chronic conditions [28]. The issues of fear, mistrust and concerns about the efficacy and side effects of the vaccines highlighted in this paper are consistent with the WHO 3Cs for vaccine hesitancy which emphasizes such factors as key drivers and determinants of vaccine uptake, in this context, the COVID-19 vaccine [29]. In addition, disinformation and misinformation have been found to also be major sources of vaccine hesitancy in high-income countries, with myths and conspiracies shared over social media found to be important sources of vaccine refusal in earlier studies (30–32). Importantly, this suggests that the issue of vaccine hesitancy is a major global health challenge that requires all stakeholders to address this issue.

We found that long wait times for vaccination were a major barrier to vaccine uptake, leading many of our study participants to abandon the entire process or refuse vaccination. This finding is consistent with previous studies which reported long queues at hospitals, health centers or vaccination centers as a major barrier to COVID-19 vaccine uptake in Ghana and India [24, 30]. Early in the rollout of the COVID-19 vaccine, Ghana used counters, mobile clinics, markets and other public places to supplement its limited number of hospitals and vaccination centers to conduct vaccination programs [31] However, these facilities were unable to accommodate the large number of people who visited them for vaccinations, resulting in long wait times. Long wait times for treatment are consistently reported in Ghana, as a persistent problem in various service delivery departments of healthcare facilities and is a major cause of patient dissatisfaction with the quality of care [32]. This indicates that the problem of long waiting times pre-dated the onset of COVID-19 vaccination. Intervention to increase vaccination rates in Ghana requires overall health system change to improve access to healthcare.

Our study participants reported that there were poor institutional measures to prioritize key populations such as the aged, health workers and PLWNCDs, and this was a major source of discouragement to be vaccinated. This is a major challenge since these key populations are at higher risk of severe illness and death from COVID-19. To achieve the goal of pandemic control in the fairest, most ethical, and efficient way possible, key population groups need to be prioritized for early vaccination.

We also found that our study participants had low proximity-based access to vaccination centers, and this led to a delay in vaccine uptake. Evidence showed that living near vaccination sites can minimize transportation barriers to getting COVID-19 vaccine [33]. Programs to improve access to vaccines should focus on improving vaccine proximity most especially for high-risk populations such as people living with chronic conditions. Another barrier to vaccine uptake, mentioned by the participants, was vaccine stockouts. This finding is consistent with a previous study in Ghana which showed that a shortage of vaccines prevented uptake among women in two regions of Ghana [24] At the start of the vaccination program in Ghana, there was delay in receiving the second dose of the AstraZeneca\/Oxford vaccine when demand for the vaccine was high [34] Efforts to minimize vaccine stockouts would be of great importance to increasing vaccine uptake among our study population and in Ghana generally.

With respect to recommendations on how to improve COVID-19 vaccine uptake, the suggestions of the participants focused broadly on education and sensitization, house-to-house vaccination strategy and improving the number of vaccination centers. As mentioned above, even though Ghana used counters, mobile clinics, markets and other public places to supplement its limited number of hospitals and vaccination centers to conduct vaccination programs, [31] subsequent vaccine roll-out programs should factor in the population size. This strategy will ensure that the supply of vaccines is much more than its demand thus eliminating major barriers to uptake.

We acknowledge two key limitations of the study. First, although we recruited participants with different NCD conditions, the overall sample was still limited and not representative of the population of PLWNCDs in Ghana during the COVID-19 pandemic. Thus, the findings should be interpreted with caution. Participants’ responses relating to vaccine uptake were based primarily on self-reporting and this has the potential to be biased, thus caution should be exercised in any attempt to interpret the data.

## Conclusion

Our study aimed to explore perceptions of the COVID-19 vaccination campaign among PLWNCDs in three diverse regions of Ghana. Although fear of side effects emerged as a reason for hesitancy, all the remaining barriers concerned deficiencies in service delivery. Compared to studies in other regions, misinformation and disinformation did not emerge as major causes of vaccine hesitancy. If policymakers can improve community-based vaccine delivery, reduce queues and waiting times, prioritize PLWNCDs and other vulnerable groups, and improve sensitization and communication—our findings suggest there will be major improvements in COVID-19 vaccine coverage in Ghana. Overall, with on-going discussions on the post-COVID-19 pandemic, we wish to emphasize that the lessons learned from the pandemic can be translated into suggestions for improving preparedness for future pandemics.

## Supporting information

S1 Appendix Inclusivity in global research. (DOCX)

S2 Appendix Experiences of COVID-19 vaccine uptake in Ghana. (DOCX)

S3 Appendix SRQR checklist. (PDF)
