# Supplementary material for: To vaccinate or not to vaccinate? Experiences of COVID-19 vaccine uptake among people living with non-communicable diseases in Ghana: A qualitative study
Source: PLOS Glob Public Health. 2024 Oct 14;4(10):e0003820. doi: 10.1371/journal.pgph.0003820 (PMC11472953; doi:10.1371/journal.pgph.0003820)
Supplement: S2 Appendix — (DOCX) [file pgph.0003820.s002.docx]

**S2 Appendix. Experiences of COVID-19 vaccine uptake in Ghana**

**Introductions**

At the beginning of the discussion the researcher should:

- Introduce themselves and ask participants to do so too.
- Thank respondent for agreeing to be interviewed.
- Explain the purpose of the discussion (objectives/introduction) and what the respondent’s participation will involve.
- Explain that people are free to contribute as they feel comfortable and that there are no wrong or right answers, and that we are interested in their own opinions and experiences.
- Attain the informed consent of the respondent to participate and have the discussion recorded.

**++++++++++++++++++++++++++++++++++++++++++++++++++++++++++++++++++++++**

1. Briefly describe your experiences of the COVID-19 pandemic
2. How did the pandemic impact your life as a person living with a chronic disease?
3. Have you received the COVID-19 vaccine?
   1. If yes, probe for the following
   2. which of the vaccine categories/types did you receive.
   3. Did you receive a full dose?
4. Can you describe your experience after receiving the full dose?
5. Do you feel people living with NCDs were given priority and adequately considered by the government in the vaccination campaigns and arrangements across the country? Explain your answer.
6. Can you explain to me the reasons for the for uptake/vaccination?
7. If you did not take the vaccine or a full dose, what accounted for this decision?
8. Did you experience or encounter any challenge in your attempt to receive or access the vaccination centres for the shots. Briefly describe your experience
9. Based on your experiences, what recommendations will you make to government to improve the national vaccination process to ensure wider coverage
10. Did you have any question to ask or any contribution to make on this topic that was not preciously captured or discussed?

**Thank you.**
